# Clinical characteristics and laboratory indicator analysis of 67 COVID-19 pneumonia patients in Suzhou, China

**DOI:** 10.1186/s12879-020-05468-8

**Published:** 2020-10-12

**Authors:** Yi Wang, Lin Yao, Jian-Ping Zhang, Pei-Jun Tang, Zhi-Jian Ye, Xing-Hua Shen, Jun-Chi Xu, Mei-Ying Wu, Xin Yu

**Affiliations:** 1 The Affiliated Infectious Hospital of Soochow University, 10, Guangqian Road, Suzhou, Jiangsu P. R. China 215000; 2 The Fifth People’s Hospital of Suzhou, Suzhou, China

**Keywords:** OVID-19, SARS-CoV-2, FIB, Treatment, Hormone

## Abstract

**Background:**

Sudden exacerbations and respiratory failure are major causes of death in patients with severe coronavirus disease 2019(COVID-19) pneumonia, but indicators for the prediction and treatment of severe patients are still lacking.

**Methods:**

A retrospective analysis of 67 collected cases was conducted and included approximately 67 patients with COVID-19 pneumonia who were admitted to the Suzhou Fifth People’s Hospital from January 1, 2020 to February 8, 2020. The epidemiological, clinical and imaging characteristics as well as laboratory data of the 67 patients were analyzed.

**Results:**

The study found that fibrinogen (FIB) was increased in 45 (65.2%) patients, and when FIB reached a critical value of 4.805 g/L, the sensitivity and specificity、DA, helping to distinguish general and severe cases, were 100 and 14%、92.9%, respectively, which were significantly better than those for lymphocyte count and myoglobin. Chest CT images indicated that the cumulative number of lung lobes with lesions in severe patients was significantly higher than that in general patients (*P* < 0.05), and the cumulative number of lung lobes with lesions was negatively correlated with lymphocyte count and positively correlated with myoglobin and FIB. Our study also found that there was no obvious effect of hormone therapy in patients with severe COVID-19.

**Conclusions:**

Based on the retrospective analysis, FIB was found to be increased in severe patients and was better than lymphocyte count and myoglobin in distinguishing general and severe patients. The study also suggested that hormone treatment has no significant effect on COVID-19.

## Background

Novel coronavirus pneumonia is an acute infectious disease caused by severe acute respiratory syndrome coronavirus 2 (SARS-CoV-2) infection and is mainly transmitted by respiratory particles [1]. Since the first novel coronavirus pneumonia case was reported in Wuhan, China, the new coronavirus spread rapidly across the country, and it is also endemic in many countries around the world, including Japan, Singapore, Thailand and the United States [2]. Thus far, thousands of cases have been confirmed. On February 11, 2020, the World Health Organization (WHO) officially announced that the cause of the new coronavirus pneumonia is a new variant of coronaviruses and named the disease that it caused as coronavirus disease 2019 (COVID-19). On February 8, 2020, the National Health Commission of China temporarily named the pneumonia caused by the new coronavirus new coronavirus pneumonia (NCP).

SARS-CoV-2 is a coronavirus that belongs to the genus *Betacoronavirus*, with an envelope and particles that are round or oval and often polymorphic, with a diameter of 60–140 nm. Its genetic characteristics are different from those of severe acute respiratory syndrome coronavirus (SARSr-CoV) and respiratory syndrome coronavirus in the middle east (MERSr-CoV) [3]. Current research shows that it has more than 85% homology with bat SARS-like coronavirus (bat-SL-CoVZC45) [4]. When isolated and cultured in vitro, SARS-CoV-2 can be found in human respiratory epithelial cells in approximately 96 h, while it takes approximately 6 days to isolate and culture in VeroE6 and Huh-7 cell lines. Thus far, we are not fully aware of the pathogenesis of COVID-19 pneumonia, its development process in the body, or its route of transmission. The gold standard for the diagnosis is real-time fluorescence RT-PCR to test whether samples are positive for the nucleic acid of SARS-CoV-2, but this method is time-consuming and has the possibility of false negatives. Understanding the early epidemiological and clinical characteristics of COVID-19 pneumonia patients is extremely important for diagnosis; therefore, we conducted a retrospective analysis of 67 cases of COVID-19 pneumonia.

## Methods

### Patients

A total of 107 cases were collected, including 20 healthy donors, 20 tuberculosis patients and 67 patients with COVID-19 pneumonia. The healthy donors were from the physical examination center and exclude tuberculosis, hepatitis B, hepatitis C, HIV infection and other pulmonary disease such as COPD, Bronchitis etc., the ages are 18 to 60. Tuberculosis patients are randomly selected inpatients with sputum culture positive about *Mycobacterium tuberculosis* of our hospital and exclude hepatitis B, hepatitis C, HIV infection. COVID-19 pneumonia patients were admitted to the Pulmonary Department Building A of Suzhou Fifth People’s Hospital from January 1, 2020 to February 8, 2020 (the diagnosis conformed to the diagnostic criteria (NHC Diagnostic Criteria (V5)) set out in the Diagnosis and Treatment of Pneumonia Infected by Novel Coronavirus (5th trial edition) issued by the General Office of the National Health Commission on February 4, 2020). According to the NHC Diagnostic Criteria (V5), 1 cases were classified as mild, 48 cases as general, 16 cases as severe, and 2 cases as fatal. Due to the limited sample size and to reduce sampling error, we combined the mild and general cases into Group A, and we combined the severe and fatal cases into Group B. We obtained informed consent from the subjects, and the study was approved by Ethics Committee of Suzhou Fifth People’s Hospital (clearance number:2020–013).

### Study inclusion criteria

COVID-19 pneumonia patients present with chest CT imaging abnormalities, even asymptomatic patients, with rapid evolution from focal unilateral to diffuse bilateral ground-glass opacities that progress to or coexist with consolidations within 1–3 weeks. The assessment of both imaging features and clinical and laboratory findings could facilitate the early diagnosis of COVID-19 pneumonia. The classification of the severity of COVID-19 conforms to the NHC Diagnostic Criteria (V5) and is set out as follows: mild type: clinical symptoms are mild and no pneumonia is present in chest CT images; general type: fever, respiratory tract and other symptoms, chest CT images show pneumonia; severe type (meets any of the following): ① respiratory distress and RR ≥ 30 breaths/min; ② oxygen saturation at rest≤93%; or ③ PaO_2_/FiO_2_ ≤ 300 mmHg; and fatal type (meets any of the following): ① respiratory failure and the need for mechanical ventilation; ②shock; or ③ the combined failure of other organs requires ICU monitoring and treatment.

### Specimen collection

Blood samples of Group A and Group B were taken within 24–48 h of admission. Blood tests were performed, and C-reactive protein, routine biochemical and coagulation parameters, and myoglobin levels were tested. In the control group, fasting venous blood samples were collected for examination on the day of the physical exam.

### Major equipment

The instrument used to analyze routine blood samples was a Sysmex-XN3000 blood analyzer from Japan Sysmex Corporation, and the reagent used was a supporting product of the company. The instrument used to detect CRP was a Jet-iStar 3000 immunoassay analyzer from Zhonghan Shengtai Biotechnology Co., Ltd., and the reagent was a supporting product of the company. The instrument used to detect the coagulation index was a CA1500 Hemagglutination Apparatus from Japan Sysmex Corporation, and the reagent was a supporting product of the company. The instrument used to detect myoglobin was a Roche 411, and the reagent was a supporting product of the company.

### Statistical processing

SPSS 31.0 statistical software was used for data processing. Measurement data for normal distributions is expressed as ±s; Comparisons between groups were performed by t test, the test level was α = 0.05 (both sides); the difference was statistically significant with *P* < 0.05; The measurement data of skewed distribution is expressed as “median (quartile) [M (Q1, Q3)]”, and the differences between groups were compared by using the rank sum test. Spearman’s correlation was used for correlation analysis. The receiver operating characteristic (ROC) curve were drawn, the area under the ROC curve (AUC) were calculated, and the absolute value of lymphocytes, fibrinogen, myoglobin and other indicators selected to alert the best cutoff value of the NCP and the corresponding sensitivity and specificity.

## Results

### Comparison of the general patient information of group a and group B

Sixty-six patients with COVID-19 pneumonia manifested chest CT imaging abnormalities, even asymptomatic patients, with rapid evolution from focal unilateral to diffuse bilateral ground-glass opacities that progressed to or coexisted with consolidations within 1–3 weeks. Combining the assessment of imaging features with clinical and laboratory findings could facilitate the early diagnosis of COVID-19 pneumonia. Sixty-seven patients were collected in the study, the average age was 46 years. There were 49 patients in Group A, with 29 males, and 18 patients in group B, with 13 males. No significant difference in sex was observed between the two groups (*P* > 0.05). Ninety percent of the patients in this study had a history of exposure to the Hubei epidemic area, with an incubation period of 2–14 days and a median incubation period of 7.0 days (4.0–10.0). Twenty-two percent of patients had chronic underlying diseases, of which hypertension and diabetes accounted for the highest proportion of chronic diseases, and there was no significant difference between the two groups (*P* > 0.05) (Table 1).

**Table 1:** Comparison of general patient information ([*M*(*Q*_1_, *Q*_3_)])

Group	All patients (*n* = 67)	Group A (*n* = 49)	Group B (*n* = 18)	*P* value
Characteristics				
Age [years, *M* (*Q*_1_, *Q*_3_)]	44.0 (36.0,59.0)	41.0 (36.0,57.0)	46.0 (37.3,60.3)	0.344
Sex	–	–	–	0.255
Men	41 (61.2%)	28 (57.1%)	13 (72.2%)	–
Women	26 (38.8%)	21 (42.9%)	5 (27.8%)	–
Exposure to the Hubei epidemic area	60 (89.6%)	43 (87.8%)	17 (94.4%)	0.435
Incubation period (days)	7 (4,10)	7.0 (4.5,10)	6.5 (4,10)	0.664
Comorbidity	15 (22.4%)	12 (24.5%)	3 (16.7%)	0.503
Hypertension	4 (6.0%)	4 (8.2%)	0	–
Diabetes	4 (6.0%)	2 (4.1%)	2 (11.1%)	–
Respiratory diseases	1 (1.5%)	1 (2.0%)	0	–
Chronic liver disease	3 (4.5%)	3 (6.1%)	0	–
Chronic kidney disease	2 (3.0%)	2 (4.1%)	0	–
Malignancy	2 (3.0%)	2 (4.1%)	0	–
Other diseases	3 (4.5%)	2 (4.1%)	1 (5.6%)	–

### Differences in patients’ clinical symptoms and imaging findings

**Fig. 1:**
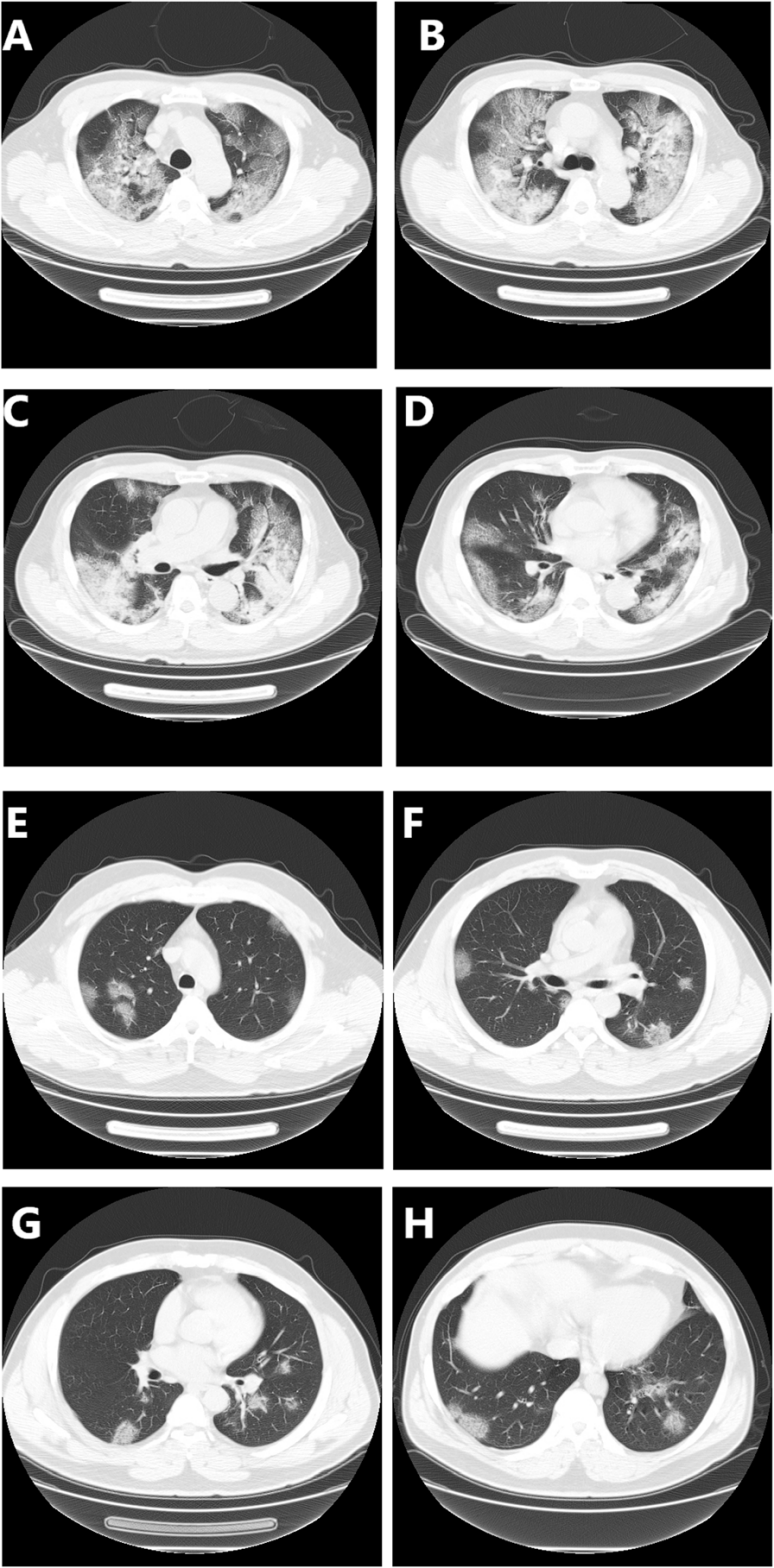
CT results of COVID-19 pneumonia patients. (**a**-**d**) CT results of severe patient;(**e**-**g**) CT results of general patient

Of the 67 patients, 91% had fever symptoms. There were more fever patients (> 38.5 °C) in Group B; the duration of fever was longer in Group B than in Group A, and the difference was statistically significant (*P* < 0.05). In addition, cough (76.1%), fatigue (61.2%) and shortness of breath (18.8%)were the most common symptoms, and a few patients had diarrhea (15%), which was more common in Group A than in Group B. According to the imaging findings, 98.5% of the patients had lung lesions, among whom the cumulative number of lung lobes with lesions in Group B was statistically significantly higher (*P* < 0.05) than that in Group A (Fig. 1). The median time for the progression of lesions in COVID-19 pneumonia patients was 3.0 days (2.0, 5.0), and there was no significant difference between the two groups (*P* > 0.05) (Table 2).

**Table 2:** Differences in patients’ clinical symptoms and imaging findings

Group	All patients (n = 67)	Group A (n = 49)	Group B (n = 18)	*P* value
Characteristics				
Clinical symptoms at admission				
Fever	61 (91.0%)	43 (87.8%)	18 (100%)	0.123
T ≤ 38.5 °C	42 (62.7%)	34 (69.4%)	8 (44.4%)	0.017
T>38.5 °C	19 (28.4%)	9 (18.4%)	10 (55.6%)	
Fever duration (days)	5.0 (2.0,9.0)	4.0 (1.0,9.0)	7.5 (4.0,10.5)	0.031
Cough	51 (76.1%)	35 (71.4%)	16 (88.9%)	0.089
chest tightness	13 (18.8%)	2 (4.1%)	11 (61.1%)	<0.001
Fatigue	41 (61.2%)	24 (49.0%)	17 (94.4%)	<0.001
Diarrhea	10 (15.0%)	9 (18.4%)	1 (5.6%)	0.131
Muscle ache	7 (10.4%)	3 (6.1%)	4 (22.2%)	0.135
Chest x-ray and CT findings				
No lung lesion	1 (1.5%)	1 (2.0%)	0	–
Lung lesions	66 (98.5%)	48 (98.0%)	18 (100%)	
Total number of lung fields in the lesion (2 lung fields on the left and 3 lung fields on the right)	3.0 (3.0, 4.0)	3.0 (2.0,4.0)	4.5 (4.0,5.0)	<0.01
Days for illness lesion progression	3.0 (2.0, 5.0)	2.0 (1.0, 5.0)	3.0 (2.0, 5.0)	0.649

### Comparison of laboratory indicators for patients

Among the 67 COVID-19 pneumonia patients, 29 patients had leukocyte abnormalities, of whom 20 (29.9%) had decreased white blood cells and 9 (13.4%) had increased white blood cells. A total of 16 (23.9%) patients had an increased neutrophil ratio, and the absolute value of lymphocyte counts was decreased in 31 (46.3%) patients (14 (28.6%) in Group A and 17 (94.4%) in Group B). The differences in the above indicators were statistically significant (*P* < 0.05). C-reactive protein increased in 33 of 67 patients, especially in Group B, and the difference was statistically significant (P < 0.05). Abnormal liver function was found in 18 (26.9%) patients (alanine aminotransferase (ALT) and aspartate aminotransferase (AST) increased in 15 and 11 patients, respectively, with the maximum value of ALT being 181 U/L and that of AST being 158 U/L). Liver function indexes in Group B were higher than those in Group A, with statistically significant differences (*P* < 0.05). There were 11 (16.4%) patients with elevated bilirubin: 5 (10.2%) patients in Group A and 6 (33.3%) patients in Group B. There was no significant difference between the two groups (*P* > 0.05). Thirty-six patients (53.7%) had a decrease in albumin. The decrease in albumin in Group B was larger than that in Group A. The difference was statistically significant (*P* < 0.05). Most patients (89.6%) had elevated lactate dehydrogenase (LDH) 43 (87.8%) patients in Group A and 17 (94.4%) patients in Group B) (P < 0.05). Only 2 patients had increased renal function. In addition, fibrinogen (FIB), D-dimer, and myoglobin were significantly increased in patients in Group B, which was significantly different from Group A (P < 0.05) (Table 3).

**Table 3:** Comparison of laboratory indicators for patients

Group	All patients (n = 67)	Group A (n = 49)	Group B (n = 18)	*P* value
Laboratory indicators				
WBC (*10^9/L, normal value 3.5–9.5)	4.5 (3.4,6.1)	4.3 (3.3,5.8)	6.2 (4.3,10.4)	0.017
<3.5	20 (29.9%)	18 (36.7%)	2 (11.1%)	–
>9.5	9 (13.4%)	2 (4.1%)	7 (38.9%)	–
N% (normal value 40–75)	63.2 (54.0,74.0)	58.0 (51.1,67.6)	79.2 (72.2,85.9)	<0.001
<40	2 (3.0%)	1 (2.0%)	1 (5.6%)	–
>75	16 (23.9%)	4 (8.2%)	12 (66.7%)	–
L(*10^9/L, normal value 1.1–3.2)	1.1 (0.8,1.4)	1.2 (1.1,1.6)	0.7 (0.6,0.8)	<0.001
<1.1	31 (46.3%)	14 (28.6%)	17 (94.4%)	–
>3.2	0	0	0	–
CRP (mg/L, normal value<10)	9.6 (2.2,21.5)	6.6 (0.8,18.0)	24.6 (8.1,43.1)	0.01
>10	33 (49.3%)	20 (40.8%)	13 (72.2%)	–
TB (umol/L, normal value 4.0–17.1)	9.7 (6.7,14.4)	9.1 (6.7,12.6)	12.4 (6.7,19.4)	0.348
<4.0	2 (3.0%)	2 (4.1%)	0	–
>17.1	11 (16.4%)	5 (10.2%)	6 (33.3%)	–
AST (U/L, normal value 8–40)	27.0 (21.0,34.0)	24.0 (21.0,31.0)	33.0 (26.3,48.3)	0.002
>40	11 (16.4%)	5 (10.2%)	6 (33.3%)	–
ALT (U/L, normal value 5–40)	29.0 (23.5,38.5)	28.0 (23.0,35.0)	37.0 (27.3, 59.0)	0.041
>40	15 (22.4%)	7 (14.3%)	8 (44.4%)	–
Albumin (g/L, normal value38–55)	37.6 (33.7,39.6)	38.3 (36.8,40.0)	32.7 (31.7,35.8)	<0.001
<38	36 (53.7%)	20 (40.8%)	16 (88.9%)	
LDH (U/L, normal value135–225)	426.0 (349.5547.5)	417.0 (337.0,499.0)	532.5 (424.8929.0)	0.009
>225	60 (89.6%)	43 (87.8%)	17 (94.4%)	–
Cr (umol/L, normal value44–106)	66.0 (55.5,82.9)	66.0 (54.3,86.3)	66.1 (57.5,77.0)	0.741
<44	7 (10.4%)	5 (10.4%)	2 (11.1%)	–
>106	2 (3.0%)	0	2 (11.1%)	–
MB (ng/mL, normal value25–58)	24.3(< 21.0,38.1)	< 21.0(< 21.0, 32.2)	40.2 (24.3,69.6)	0.02
<25	36 (53.7%)	30 (61.2%)	6 (33.3%)	–
>58	3 (4.5%)	3 (6.1%)	0	–
FIB (g/L, normal value2–4)	4.5 (3.8,5.4)	4.1 (3.5,4.6)	6.4 (5.7,7.0)	<0.001
>4	45 (67.2%)	27 (55.1%)	18 (100%)	–
D-dimer (ug/L, normal value0–550)	220 (160,375)	200 (150,270)	375 (242.5832.5)	0.037
>550	10 (14.9%)	3 (6.1%)	7 (38.9%)	–

### Comparison of lymphocyte count and myoglobin detection in patients

In a comparison of patients in Group A with patients in Group B, the lymphocyte count absolute value (L), myoglobin (MB) and fibrinogen of COVID-19 pneumonia patients in Group A were significantly higher than those in Group B, with statistically significant differences (*P* ≤ 0.001) (Table 4). In a comparison of Group A with the healthy control group, the L was significantly higher in Group A than in the healthy control group (*P <* 0.001). Compared with group A, there was no significant difference in the absolute value of lymphocytes and fibrinogen in the tuberculosis group (*P* > 0.05) (Table 4).

**Table 4:** Comparison of lymphocyte and myoglobin detection in patients

Indicators	L(*10^9)	MB	FIB
Group			
Group A (n = 49)	1.23a(1.09,1.63)	<21a(<21,32.57)	4.11a(3.10,4.58)
Group B (n = 18)	0.71 (0.55,0.81)	41.27 (23.76,76.61)	6.42 (5.60,7.20)
Healthy control group (*n* = 20)	1.96b(1.48,2.35)	–	–
Tuberculosis group (n = 20)	1.63c(1.29,1.86)	–	4.25c(3.11,4.72)

Note: comparing with Group B, ^a^*p*<0.001 for indicators in Group A, ^b^*p*<0.001 for indicator of L in healthy control group, comparing with healthy control group, *p*<0.05 for indicator of L in comparing with tuberculosis group, comparing with Group A, ^c^*p*>0.05 for indicators in tuberculosis group

### ROC curve analysis

**Fig. 2:**
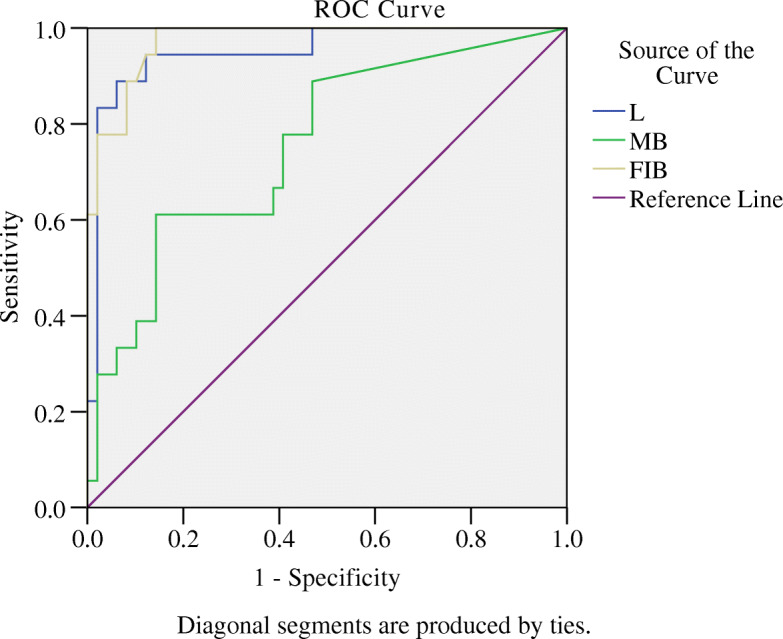
ROC curve of lymphocyte absolute value (L), myoglobin (MB), fibrinogen (FIB)

With the data of both Group A and Group B plotted in a receiver operating characteristic (ROC) curve, the data of Group A and Group B were compared. The ROC curve was used to evaluate the L, MB, FIB and other indicators for the prediction of severe disease in patients with COVID-19 pneumonia (Fig. 2). The optimal truncation values (with maximum Youden index) were selected, and the optimal truncation values of L, MB and FIB were calculated as 1.071*10^9/L, 36.6 ng/mL and 4.805 g/L, respectively. The area under the ROC curve (AUC) for L was 0.951, for MB was 0.754 and for FIB was 0.973. When the optimal truncation value of the FIB index was selected, the sensitivity, specificity and DA for the prediction of severe disease in COVID-19 pneumonia patients were 100%, 14.0 and 92.9%, respectively, and the sensitivity, specificity and DA of FIB were significantly higher than those of L and MB (Table 5).

**Table 5:** Comparison of AUC, truncation value, sensitivity, and specificity of 3 indicators

Indicator	AUC	Diagnostic threshold	Sensitivity (%)	Specificity (%)	PPV(%)	NPV(%)	DA(%)	95% confidence interval ^b^	Standard error	Significance level P (area = 0.5)
L	0.951	1.071*10^9/L	88.9	6.1	93.6	89.4	91.4	0.892–1.000	0.03	<0.0001
MB	0.754	36.6 ng/mL	61.1	14.3	81.1	68.8	73.4	0.622–0.886	0.067	0.002
FIB	0.973	4.805 g/L	100	14.3	87.5	1	92.9	0.943–1.000	0.016	<0.0001

### Correlation analysis

An analysis of the correlation of the cumulative number of lung lobes with lesions with L, MB, and FIB in 67 COVID-19 pneumonia patients indicated that the cumulative number of lung lobes with lesions was positively correlated with MB and FIB and negatively correlated with L (Table 6).

**Table 6:** Correlation of cumulative lung field number with L, MB, and FIB indexes

Parameter	Pearson Correlation coefficient	*P* value
L	−0.368	0.002
MB	0.324	0.008
FIB	0.563	<0.001

### Comparison of treatment differences

**Fig. 3:**
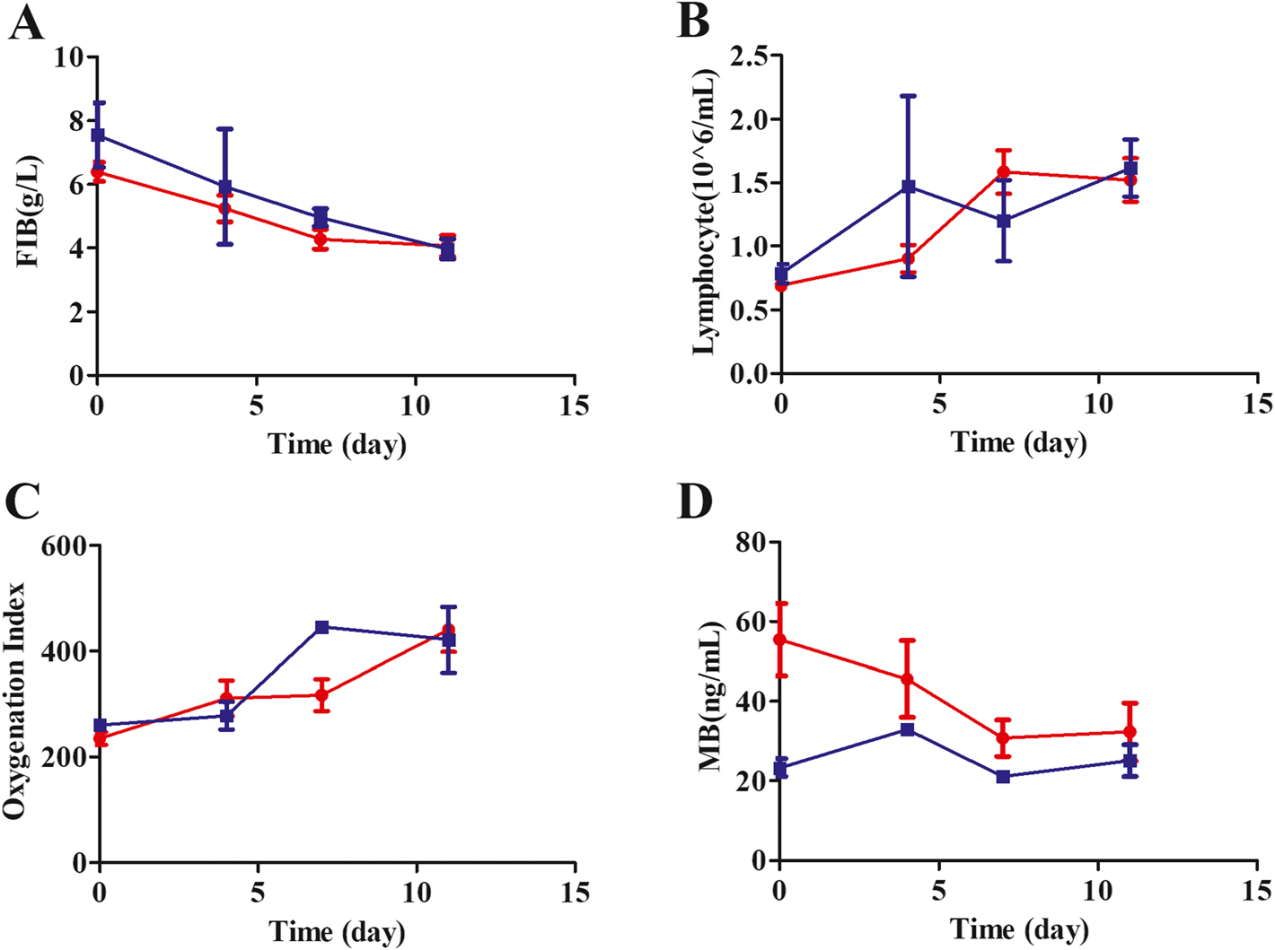
Effect of hormone therapy on oxygenation index, Lymphocyte, MB and FIB. **a** Effect of hormone therapy on FIB; **b** Effect of hormone therapy on Lymphocyte; **c** Effect of hormone therapy on oxygenation index; **d** Effect of hormone therapy on MB. Red: Hormone therapy group, Blue: Non-hormonal treatment group

Sixty-one (88.4%) patients were treated with oxygen therapy, among which nasal catheter oxygen was the main treatment in Group A, while noninvasive ventilator-assisted ventilation or high-flow oxygen was needed for some patients in Group B who were in respiratory failure, and the difference was statistically significant (*P* < 0.05). Only 22 (32.8%) patients were treated with hormone therapy, of whom the proportion in Group B was larger; the time of hormone use was longer in Group B than in Group A, and the difference was statistically significant (P < 0.05). The vast majority (97.1%) of patients received antiviral treatment immediately after admission, with no statistically significant difference between the two groups (*P* > 0.05) (Table 7). At the same time, we also analyzed the effects of hormone therapy and nonhormonal therapy. Compared with nonhormonal therapy, hormone therapy did not promote key indicators (Fig. 3).

**Table 7:** Comparison of treatment differences

Group	All patients (n = 67)	Group A (n = 49)	Group B (n = 18)	*P* value
Treatment				
Oxygen therapy	61 (91.0%)	43 (87.8%)	18 (100%)	0.044
Nasal catheter oxygen	49 (73.1%)	43 (87.8%)	6 (33.3%)	–
Mask oxygen	7 (10.4%)	0	7 (38.9%)	–
High flow oxygen	3 (4.5)	0	3 (16.7%)	–
Non-invasive ventilator	2 (3.0%)	0	2 (11.1%)	–
Glucocorticoids	22 (32.8%)	6 (12.2%)	16 (88.9%)	<0.001
Glucocorticoids using time	6.5 (5.0,9.0)	7.0 (5.0,9.0)	6.0 (5.3,8.3)	< 0.001
Antiviral therapy	67 (100%)	49 (100%)	18 (100%)	0.397

## Discussion

Current research indicates that SARS-CoV-2 is more than 85% homologous to bat-SL-CoVZC45 [5]. Therefore, it is considered that SARS-CoV-2 was transmitted by bats [6], which needs further research confirmation. For describing the clinical and laboratory characteristics and analysis the oxygen therapy and hormone therapy, we conducted a retrospective analysis of 67 cases of COVID-19 pneumonia in 2020 Suzhou, China.

This study included 67 patients with COVID-19 diagnosed in the Suzhou Fifth People’s Hospital, which shows that, similar to Chen’s study [7], patients with SARS-CoV-2 infection were mainly middle-aged and elderly individuals, with a median age of 44 years (36.0 59.0), and 42 (61.2%) were males. Ninety percent of the patients had a history of exposure to the Hubei epidemic area, with an incubation period of 2–14 days and a median incubation period of 7.0 days (4.0–10.0), which is also similar to Wang’s study [8, 9]. A total of 22.4% of patients had underlying chronic diseases, of which hypertension and diabetes accounted for a higher proportion of chronic diseases, and there was no significant difference between the two groups (*P* > 0.05).

Fever is the most common clinical symptom of COVID-19 pneumonia, with 61 (91.0%) cases in 67 patients observed. Fever occurred in the early stage of the disease, and 42 (62.7%) patients had a body temperature ≤ 38.5 °C, which was more common in Group A. Ten patients (55.6%) had a body temperature ≥ 38.5 °C in Group B, which was statistically significantly higher (*P* < 0.05) than that in Group A. In addition, the duration of fever in Group B was significantly longer than that in Group A; the median fever duration in Group B was 7.5 days (4.0–10.5), and the difference was statistically significant (P < 0.05). Cough (76.1%), fatigue (61.2%) and shortness of breath (18.8%) were also common, among which shortness of breath was more common in Group B, while fatigue was more common in Group A, and the differences were statistically significant (P < 0.05). These symptoms are considered to be related to lung lobe invasion in severe patients. Diarrhea (15.0%) and muscle soreness (10.4%) were less common in patients; however, we still need to be alert to the patients who are diagnosed with mainly gastrointestinal symptoms, pay attention to strengthening protection, and conduct timely SARS-CoV-2 nucleic acid testing for patients with a history of epidemiology.

The retrospective analysis of 67 patients with COVID-19 pneumonia indicated that 66 (98.5%) patients showed lung lesions on CT chest images, with multiple sites of distribution, and lesions were seen in both lungs and subpleural areas [10], mostly showing ground-glass opacities, consolidation, interstitial changes, and interlobular septal thickening [11, 12] (Fig. 1). The median time from illness onset to lesion progression in COVID-19 pneumonia patients was 3.0 days (2.0, 5.0). The median number of cumulative lung lobes with lesions was 3.0 (3.0, 4.0), and the cumulative number of lung lobes with lesions in Group B was significantly higher than that in Group A, with a statistically significant difference (*P* < 0.05). Laboratory data showed that the WBC, N%, and CRP in Group B COVID-19 pneumonia patients were significantly higher than those of Group A patients, and the differences were statistically significant (P < 0.05). The increase in these levels is considered to be caused by systemic inflammation, which was relatively obvious in severe patients, but the possibility of bacterial infection or secondary fungal infection in some severe patients could not be ruled out. The absolute value of lymphocyte counts decreased significantly in 46.3% of COVID-19 pneumonia patients, especially in Group B, compared with Group A, with a statistically significant difference, suggesting that cellular immune function decreased in the early stage in COVID-19 pneumonia patients, especially in severe patients. In addition, the L in the group A and tuberculosis group has no significantly different, but tuberculosis mainly causes the reduction of CD4^+^T cells, while the COVID-19 is CD8^+^T cells. Although the performance of them is similar, the types of lymphocytes which decrease are different. A total of 18 (26.9%) patients had abnormal liver function (ALT maximum value of 181 U/L, AST maximum value of 158 U/L). The liver function index of Group B was statistically significantly higher (*P* < 0.05) than that of Group A, suggesting that severe patients are more likely to have liver dysfunction. Most patients had normal renal function, and only 2 (3.0%) had abnormal renal function indicators, both of whom were severe patients, suggesting that SARS-CoV-2 may not cause significant kidney damage. Thirty-six (53.7%) patients had hypoproteinemia, and serum albumin (ALB) in Group B was significantly lower than that in Group A, suggesting that the function of synthetic ALB was decreased by liver function damage in severe patients. In addition, the basal metabolic rate and resting energy consumption of severe patients were high, and ALB catabolic metabolism was accelerated. Therefore, attention should be paid to the treatment of decreased albumin levels in severe patients. FIB is a coagulation factor mainly secreted into the blood by liver cells. It is involved in the blood coagulation process and is a key factor in thrombosis. In addition, FIB is also a stress response protein FIB [13]. A total of 45 (67.2%) patients had elevated blood FIB content, suggesting that SARS-CoV-2 infection could lead to a stress response in the body, promote the synthesis and release of FIB by liver cells and macrophages, and thereby increase serum. In addition, FIB in Group B was significantly higher than that in Group A (*P* < 0.05). The increase in FIB in COVID-19 patients is considered to be caused by systemic inflammation and is relatively obvious in severe patients. D-dimer is a product of fibrinolytic cross-linked fibrin clot formation. Elevated D-dimer levels indicate high blood clotting and are a sensitive marker of acute thrombosis. This study shows that the value of D-dimer in Group B was significantly higher than that in Group A (P < 0.05). It is considered that harmful substances such as viruses and endotoxins can activate coagulation factor XII after entering the blood, activate the endogenous coagulation system, and activate the fibrinolytic system, which leads to an increase in D-dimer. Severe patients often have systemic inflammation, which could cause endothelial function to be impaired, resulting in platelet aggregation and the release of coagulation factors, thereby leading to the hyperfunction of the fibrinolytic system. The increase in FIB and D-dimer indicates that preventive anticoagulation therapy should be given to COVID-19 pneumonia patients, especially severe patients.

Treatment results indicated that 61 (91.0%) patients were treated with oxygen therapy, among which nasal catheter oxygen was the main treatment (43 (87.8%) patients in Group A, 6 (33.3%) patients in Group B). Some patients in Group B were associated with respiratory failure; thus, noninvasive ventilator-assisted ventilation or high-flow oxygen was needed, and the difference was statistically significant (*P* < 0.05). All patients received antiviral treatment immediately after admission, while only 22 (32.8%) patients were treated with hormone therapy, most of whom were from Group B. Additionally, the time of hormone use was statistically significantly longer (P < 0.05) in Group B than in Group A.

In this study, L, MB and FIB were selected as the meaningful laboratory indicators to help distinguish between general and severe COVID-19. The results showed that the values of L, MB, and FIB in Group B were statistically significantly different (P < 0.05) from those in Group A. When FIB reached a critical value of 4.805 g/L, the sensitivity and specificity were 100 and 14.3%, respectively, which were significantly better than those of L and MB. It can be seen from the ROC curve that FIB had the largest area under the ROC curve (0.973), indicating that FIB could be used as an effective laboratory indicator to help distinguish general and severe COVID-19, but the specificity of FIB was low. Therefore, a comprehensive diagnosis should be made based on clinical manifestations and meaningful data. For patients with chest tightness, L < 1.071*10^9/L, and FIB significantly higher than 4.805 g/L, we should be alert to the possibility that they may subsequently progress into severe COVID-19 or have severe tendencies, which will help in the timely clinical assessment of the condition and the adjustment of treatment.

Our study has some limitations. This study did not cover all the COVID-19 patients in our hospital, some patients were excluded but were not diagnosed, the number of selected patients was relatively small, and there might be biasing factors in the case selection. Therefore, the findings of statistical tests and *p* values should be interpreted with caution, and it is important to note that nonsignificant p values do not necessarily rule out the difference between Group A and Group B patients. In addition, the patients’ symptoms of discomfort are highly subjective; therefore, there might be errors in the reporting of clinical symptoms. Some patients did not seek medical treatment in time; thus, the imaging performance may be lagging. Therefore, further research is needed to obtain a full picture of COVID-19 pneumonia.

## Conclusions

Our study demonstrates the clinical features of patients with severe and mild disease. Patients with severe disease higher peaks of fever, longer periods of fever and more lung lesions. We also found that FIB would be a better marker for indicating the progression of this disease, and with better sensitivity and specificity than lymphocyte counts and myoglobin tests. This study further characterizes the clinical features of COVID-19 pneumonia patients and shows that FIB would be a potential clinical predictor for COVID-19 patients.

## Data Availability

The datasets used and/or analysed during the current study are available from the corresponding author on reasonable request.

## Abbreviations

SARS-CoV-2: Syndrome coronavirus 2
COVID-19: Coronavirus disease 2019
NCP: New coronavirus pneumonia
FIB: Fibrinogen
L: Lymphocyte absolute value
MB: Myoglobin
